# H_2_ Production by Methane Oxy-Reforming: Effect of Catalyst Pretreatment on the Properties and Activity of Rh-Ce_0.5_Zr_0.5_O_2_ Synthetized by Microemulsion

**DOI:** 10.3390/nano13010053

**Published:** 2022-12-22

**Authors:** Jacopo De Maron, Rodolfo Mafessanti, Pio Gramazio, Elisabetta Orfei, Andrea Fasolini, Francesco Basile

**Affiliations:** 1 “Toso Montanari” Industrial Chemistry Department, Alma Mater Studiorum, Università di Bologna, 40136 Bologna, Italy; 2 Center for Chemical Catalysis—C3, Alma Mater Studiorum, Università di Bologna, 40136 Bologna, Italy

**Keywords:** oxy-reforming, Rh-Ce_0.5_Zr_0.5_O_2_, hydrogen, catalytic bed temperature profile, microemulsion

## Abstract

Green hydrogen introduction in hard-to-abate processes is held back by the cost of substituting steam reforming plants with electrolyzers. However, green hydrogen can be integrated in properly modified reforming processes. The process proposed here involves the substitution of steam reforming with oxy-reforming, which is the coupling of the former with catalytic partial oxidation (CPO), exploiting the pure oxygen coproduced during electrolysis to feed CPO, which allows for better heat exchange thanks to its exothermic nature. With the aim of developing tailored catalysts for the oxy-reforming process, Ce_0.5_Zr_0.5_O_2_ was synthetized by microemulsion and impregnated with Rh. The Ce-based supports were calcined at different temperatures (750 and 900 °C) and the catalysts were reduced at 750 °C or 500 °C. Tuning the calcination temperature allowed for an increase in the support surface area, resulting in well-dispersed Rh species that provided a high reducibility for both the metal active phase and the Ce-based support. This allowed for an increase in methane conversion under different conditions of contact time and pressure and the outperformance of the other catalysts. The higher activity was related to well-dispersed Rh species interacting with the support that provided a high concentration of surface OH* on the Ce-based support and increased methane dissociation. This anticipated the occurrence and the extent of steam reforming over the catalytic bed, producing a smoother thermal profile.

## 1. Introduction

The urgent need to shift the present energy production system toward a renewable one based on hydrogen has already been pointed out by several organizations [1,2]. Renewable hydrogen exploitation should start from those processes that already utilize it, such as ammonia production or chemical upgrading. However, using hydrogen obtained from renewable resources (green hydrogen, 3.6–7.0 €/kg) is still more expensive than employing H_2_ produced from natural gas steam reforming (1.5–3.7 €/kg) (Equation (1)) [3]. Thus, processes that require hydrogen are usually integrated with an on-site hydrogen production system by steam reforming of methane [4]. A greener alternative to methane reforming would be found in electrolysis (Equation (4)), which is a mature technology but one that still requires a high amount of energy to break water molecules to produce hydrogen and oxygen [5]. Moreover, large-scale reforming plants are highly integrated, which makes the introduction of only relative amounts of green hydrogen possible without loss of capital investment [6]. Furthermore, the major cost of hydrogen production relies on the investment cost of the hydrogen production plant [7]. For this reason, immediately replacing steam reforming plants with electrolyzers fed with renewable energy is not a market-driven process and may be opposed by industries because the high investment cost must be compensated with years of operation to generate a return [7]. The introduction of an electrolyzer that is temporarily responsible for only a smaller part of hydrogen production (green hydrogen) and the implementation of carbon capture and storage systems (CCSs) for CO_2_ co-produced from methane (blue hydrogen) can be a stepping stone to the introduction of renewables in hydrogen production processes and abate CO_2_ release. This would help to exploit the present relatively low availability of renewable energy while gaining knowledge of the potentialities and optimization of electrolyzers, removing the barriers that may be perceived by investors [8].

In a further integrated vision, the oxygen produced from electrolysis can be used to feed the reformer and increase its energy efficiency by carrying out an exothermic reaction [9,10]. Hydrogen can also be produced by catalytic partial oxidation (CPO) (Equation (2)), an exothermic process in which methane is reacted with oxygen to give hydrogen and carbon monoxide.

Steam Reforming (SR), CH_4_ + 3 H_2_O ⇆ 3 H_2_ + CO, ΔH° = +206 kJ/mol (1)

Catalytic Partial Oxidation (CPO), CH_4_ + 0.5 O_2_ ⇆ 2 H_2_ + CO, ΔH° = −36 kJ/mol (2)

Water Gas Shift (WGS), CO + H_2_O ⇆ H_2_ + CO_2_, ΔH° = −41 kJ/mol (3)

Electrolysis, H_2_O ⇆ H_2_ + 0.5 O_2_ (4)

Great hurdles to industrial CPO are the cost of oxygen purification from air and the high exothermicity of the reaction. The first can be avoided by using pure O_2_ produced from water electrolysis, the latter by coupling exothermic CPO with endothermic steam reforming. Steam reforming and CPO are subjected to highly endothermic or exothermic temperature profiles when run alone. Smaller temperature differences occur when the two processes are coupled using sub-stoichiometric O_2_ in the so-called oxy-reforming process, where the endothermic steam reforming is fed by the exothermic oxidation [11]. Recent studies have shown that the integration of steam reforming and CPO at low temperatures allows for reduced oxygen consumption (O_2_/C below 0.3) compared to classical CPO while increasing the energy efficiency of the whole process [12]. Thus, oxy-reforming can operate at lower temperatures than steam reforming (e.g., 750 °C), and with steam to carbon ratios (S/C) and O_2_/C ratios as low as 0.7 and 0.2, respectively, the cost of water evaporation and the amount of natural gas needed to heat the process are reduced [9,10,13]. Oxy-reforming is thus suitable for the integration of green hydrogen from electrolysis, as it allows the exploitation of the reforming infrastructure while taking advantage of the inexpensive pure oxygen stream and increasing the heat development and recovery.

Thus, for the best integration of hydrogen production by reforming/CPO and electrolysis, the moderate hydrogen and oxygen production of the latter should be considered, for the reasons explained above.

However, reaction conditions and catalyst design are key factors in oxy-reforming success. The present work focuses on this topic, with the aim of providing guidelines for catalyst design and development in the frame of hydrogen production. The overall oxy-reforming process is a complex combination of different reactions, mass transfer oxidation reactions occurring mainly in the first part of the reactor and endothermic reforming reactions along the whole bed, with a thermal profile resulting from their occurrence. The low content of oxygen with respect to classical CPO was chosen on the basis of previous work to limit the sharp increase in temperature at the beginning of the bed and the formation of hot spots [11,14,15,16,17,18]. Ni-based catalysts are mostly employed in reforming due to their relatively low costs [11,19,20,21,22,23]; however, compared to noble metals, they display lower steam reforming activity and are subjected to faster deactivation caused by sintering and carbon formation [11]. Rh has been also considered as an active phase for oxy-reforming [9,10,24,25,26,27,28] and its high activity in both CPO and steam reforming may fulfil the desired aim of increasing the coupling of the two reactions. In addition, increased steam reforming activity and high stability with respect to carbon formation were observed using Ce-based supports thanks to the oxygen-storage capacity of Ce and its action as a water dissociation site [9,19,29,30,31,32,33,34]. Thus, Ce-based materials have also been used as supports in reforming processes [32,35,36,37,38].

For this reason, Rh-based catalysts supported on Ce_0.5_Zr_0.5_O_2_ supports synthesized by microemulsion [9] were applied to oxy-reforming, and the effects of calcination temperature (750 °C or 900 °C) and reduction temperature (500 °C or 750 °C) on catalyst properties and activities were studied. Steam reforming activity was also investigated by carrying out low-temperature steam reforming (LTSR) tests (350–500 °C).

## 2. Materials and Methods

### 2.1. Synthesis of the Rh/Ce_0,5_Zr_0,5_O_2_ Catalysts

The Ce-Zr oxide supports were synthesized by microemulsion synthesis, as reported previously [9,33]. A 0.5 M aqueous solution of ZrO(NO_3_)_2_ × 6 H_2_O (99.00%, Sigma-Aldrich, St. Louis, MO, USA) and Ce(NO_3_)_3_ × 6 H_2_O (99.99%, Sigma-Aldrich, St. Louis, MO, USA) in a 1:1 molar ratio was prepared. Then, a water-in-oil inverse microemulsion was created by mixing 8 wt% of this aqueous solution with 58 wt% of *n*-heptane (solvent, 99.00%, Sigma-Aldrich, St. Louis, USA), 15 wt% of 1–hexanol (co-surfactant, 99.00%, Sigma-Aldrich) and 19 wt% of a non-ionic surfactant (Triton X−100, 99.00%, Sigma-Aldrich, St. Louis, MO, USA). This was mixed under stirring with another emulsion, where the Ce and Zr precursors were substituted by a base, namely, 1.5 M tetramethylammonium hydroxide pentahydrate (TMAH = (CH_3_)_4_NOH × 5 H_2_O, 97.00%, Sigma-Aldrich, St. Louis, MO, USA). The resulting mixture was aged at room temperature for 4 h, then filtered and washed with methanol. The fine powders obtained by microemulsion were thermally treated to obtain the Ce-Zr oxides. These were then calcined at 750 °C and 900 °C at 2 °C/min for 5 h. Incipient wetness impregnation (IWI) was used to deposit an amount of 2.7% of Rh (weight of metal over weight of support), as previously reported [9]. Rh (III) nitrate solution: ~10 wt% Rh in >5 wt% in HNO_3_ (Sigma Aldrich) was used as a precursor. The deposition was completed by drying at 120 °C for 2 h and calcination at 500 °C (ramp rate of 2 °C/min) for 5 h.

Pellets in the range of 14–20 mesh were obtained from the calcined powders by crushing over sieves.

### 2.2. Catalyst Characterizations

A Philips PW1050/81 diffractometer equipped with a graphite monochromator in the diffracted beam and controlled by a PW1710 unit (Cu Kα, λ = 0.15418 nm) was used for X-ray diffraction (XRD) analysis, which ranged between 20° and 80° (2θ) at 0.2°/s. Average crystallite size (CS) was assessed using Equation (5) (Scherrer equation).

CS = (Kλ)/(βcosθ); [K = shape factor = 0.9; β^INST^ = 0.07; β = (β^exp^ − β^INST^)] (5)

An ASAP 2020 Micromeritics sorptiometer was used for nitrogen adsorption–desorption isotherms. The data were analyzed using software with standard BET and BJH methods. The nitrogen gas purity was 99.999%. The samples were pre-treated at 150 °C and 30 mmHg for 30 min and then heated up to 250 °C for 30 min.

Temperature-programmed reduction–oxidation (TPRO) cycles were performed to study the redox properties of the catalysts with the Micromeritics Autochem 2920 instrument. All the samples (0.06–0.1 g) were pre-treated with flowing He (30 mL/min) while being heated to 150 °C. Then, the samples were cooled down to 50 °C and TPR/O/R cycles were carried out by fluxing 5% H_2_/Ar (30 mL/min) while being heated from 60 °C to 950 °C at a rate of 10 °C/min. An isotherm at this final temperature of 30 min was performed. He was sent, the temperature was lowered to reach 50 °C and TPR was followed by temperature-programmed oxidation (TPO). In this case, 5% O_2_/He (30 mL/min) was used and the same temperature cycle of TPR was performed. After TPO, another TPR was performed.

TEM analyses were carried out using a TEM/STEM FEI TECNAI F20 microscope combined with energy dispersive X–ray spectrometry (EDS) at 200 keV. A small amount of sample was suspended in ethanol and treated with ultrasound for 15 min. The suspension was deposited on a “multifoil-carbon film” sustained by a Cu grid, then dried at 100 °C. EDS was used to evaluate the elementary analysis of the samples.

### 2.3. Experimental Set-Up and Description of the Catalytic Tests

The catalysts were tested in a fixed-bed reactor which consisted of a tubular INCOLOY 800HT reactor (length, 500 mm; internal diameter, 10 mm) placed in a programmable furnace. A sliding thermocouple was used to control the catalytic bed temperature. Volumes of 0.5 cm^3^ of catalyst (14–20 mesh) were charged and the reduction to metallic rhodium was performed by fluxing a 500 mL flow of a H_2_/N_2_ (10:90 *v*/*v*) gas mixture at 750 °C for 12 h. A JASCO HPLC pump was used to feed deionized water which was fully vaporized before mixing with preheated oxygen. The effluent wet gaseous mixture passed through a condenser at about 0 °C to remove water vapor before the GC analysis. The dry gas (DG) was analyzed using an Agilent 490 microgas chromatograph equipped with two different columns. A 20 m long MS5A column (carrier: N_2_) separated hydrogen, while a CO_x_ 1 m long column (carrier: He) was used for CH_4_, CO and CO_2_. Both modules were furnished with a TCD detector.

The feed composition was set to obtain a reagent mixture with a sub-stoichiometric ratio of H_2_O and O_2_, namely, an O_2_/C ratio of 0.21 and a S/C ratio of 0.70.

The equilibrium compositions and conversions were calculated with CEA-NASA software.

An average catalytic bed temperature was used for the calculations.

The conditions employed in the catalytic tests are reported in Table 1.

CH_4_ conversion was taken as a reference to evaluate the activities of the tested catalysts and was compared with that calculated at the thermodynamic equilibrium.

Experimental methane conversions were calculated based on the vol. % of products and unreacted methane obtained from GC analysis and were expressed according to Equation (6):

X = (% CO_(OUT)_ + % CO_2 (OUT)_)/(% CH_4 (OUT)_ + % CO_(OUT)_ + % CO_2 (OUT)_) × 100 (6)

Carbon balances over 95% were obtained in most of the tests.

## 3. Results and Discussion

### 3.1. Catalyst Characterization

#### 3.1.1. X-ray Diffraction Analysis

**Figure 1:**
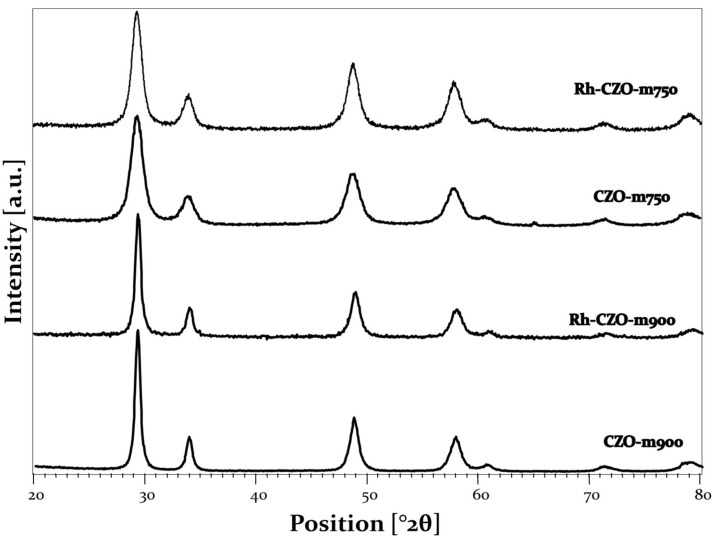
XRD analysis of (from top to bottom) Rh-CZO-m750, CZO-m750, Rh-CZO-m900 and CZO-m900.

The CeZr supports obtained by microemulsion were analyzed using XRD analysis. The XRD characterization of the CeZr oxide was reported in a previous work, where it was demonstrated that the microemulsion synthesis provided effective catalyst support for the oxy-reforming reaction [9]. Therein, we reported that a homogeneous Ce_0.5_Zr_0.5_O_2_ (CZO) phase was obtained after calcination, which is difficult to obtain with other techniques [9]. This is an advantage of employing microemulsion synthesis. The samples calcined at different temperatures all showed the presence of a mixed oxide phase related to the formation of Ce_0.5_Zr_0.5_O_2_ (Figure 1 and Figure S1). Increasing calcination temperature also increased the crystallinity of the synthesized samples (Figure S1). Crystallite average sizes increased from 4 nm at 500 °C (Figure S1) to 8 nm at 750 °C to 14 nm at 900 °C, as calculated using Equation 5. Further increasing calcination temperature led to segregation, with the sample calcined at 1000 °C showing an enlargement of the reflection caused by the presence of two superimposed peaks and two different phases at 1100 °C (one enriched in Ce and one enriched in Zr). Following these results, the samples calcined at 750 °C and 900 °C were selected for the catalytic application. In fact, a calcination at 500 °C would have provoked a structural change in the catalytic tests that were carried out at 750 °C, while at temperatures higher than 1000 °C the segregation of the phases would have led to a less homogeneous catalyst. XRD analysis was carried out, also, after the impregnation of the Rh nitrate precursor, followed by calcination at 500 °C (Figure 1).

In this case, only the Ce_0.5_Zr_0.5_O_2_ phase was again displayed, while no phase related to Rh species was shown, as the produced Rh oxide particles were well dispersed and present in low wt. %. Nevertheless, the average crystallite dimension increased for Rh-CZOm900 from 14 to 20 nm, while it did not change for Rh-CZO-750. This slight enlargement could be related to the second calcination treatment carried out after Rh impregnation.

#### 3.1.2. Nitrogen Physisorption Analysis

The morphological properties of the samples were analyzed through nitrogen physisorption analysis (Table 2).

CZOm750 was characterized by a surface area of 54.1 m^2^/g, which decreased to 14.5 m^2^/g when the calcination temperature was raised to 900 °C, due to sintering of the oxide particles. Isothermal linear plots (Figure S2) of the bare sample show a type IV curve, indicative of mesoporous materials and a higher amount of adsorbed nitrogen for the CZOm750 sample, thanks to its surface area. The two samples show different hysteresis patterns in the adsorption plots, suggesting different porosities. In fact, the pore distribution graph (Figure S3) displays a pore distribution centered around 5 nm for CZOm750 and one around 20 nm for CZOm900. The analysis was also carried out on the impregnated catalysts before reduction, i.e., on the catalysts in the form in which they are charged in the reactor. Even in this case, surface area decreased from 34.6 m^2^/g to 11.5 m^2^/g for the samples calcined at 750 °C and 900 °C, respectively. The isothermal linear plots (Figure S4) were similar to those of the bare supports, indicating that meso-porosity was kept and that pore distribution was also close to the bare supports (Figure S5). However, impregnation decreased total surface area due to slight sintering of the nanoparticles, as shown by XRD and caused by the calcination carried out after impregnation.

#### 3.1.3. Temperature-Programmed Reduction–Oxidation

**Figure 2:**
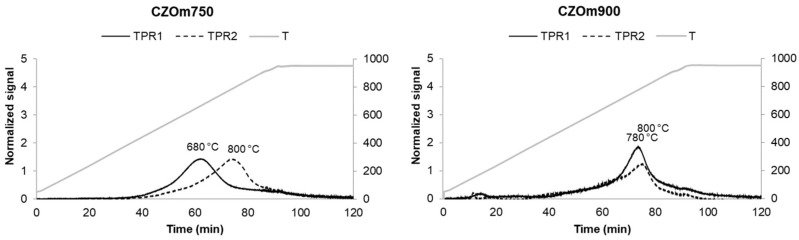
Temperature-programmed reduction of CZOm750 and CZOm900 samples. The dotted line represents the temperature-programmed reduction carried out after temperature-programmed oxidation.

Temperature-programmed reductions and oxidations were carried out on both bare supports and impregnated catalysts, results are reported in Table 3. Bare CZOm750 displayed a broad peak centered at 680 °C, while CZOm900 displayed one centered at 780 °C (Figure 2), suggesting that the redox properties of the support increased at lower calcination temperatures. The presence of only one broad peak for Ce_0.5_Zr_0.5_O_2_ has been previously reported in the literature and has been related to high oxygen mobility in the bulks for such oxides [39]. The higher surface area (Table 2) makes Ce atoms more available for reduction by gaseous hydrogen in the case of CZOm750. After an oxidation cycle (TPO), similar reduction behavior was observed, and the maxima of the reduction peaks increased as the samples were submitted to high temperatures (950 °C) during the reduction–oxidation cycles. This led to sintering of the oxide with a concurrent decrease in redox properties due to less contact with hydrogen.

**Figure 3:**
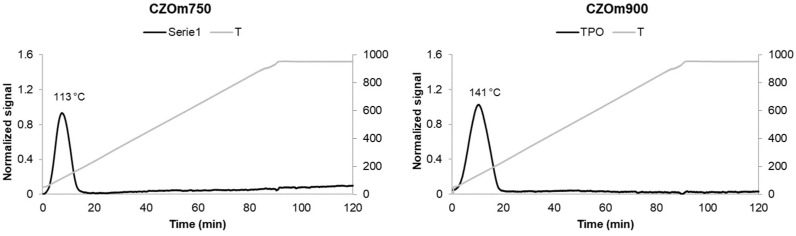
Temperature-programmed oxidation of CZOm750 and CZOm900 samples.

The oxidation peaks obtained after the first reduction are shown in Figure 3. Again, CZOm750 shows an oxidation peak at a lower temperature that indicates better redox properties due to the higher surface area of this sample. In this case, the difference is smaller, as oxidation is a fast process favored on both supports. However, the support calcined at the lower temperature was more easily reduced and oxidized, indicating a higher reactivity.

**Figure 4:**
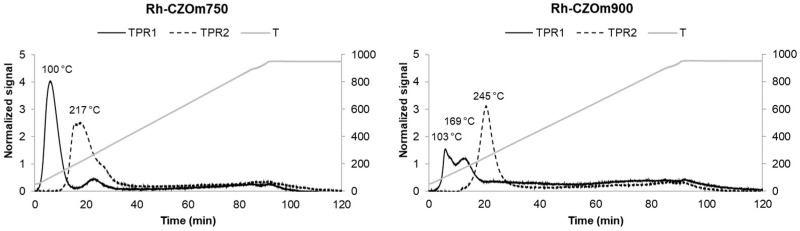
Temperature-programmed reduction of Rh-CZOm900 and Rh-CZOm750. The dotted line represents the temperature-programmed reduction carried out after temperature-programmed oxidation.

After impregnation of Rh over the two supports, TPOR was carried out again (Figure 4). The presence of the noble metal strongly decreased the reduction temperature of Ce thanks to the high reducibility of Rh and to the spillover effect of by this metal, which favors Ce reduction [40,41,42]. Rh-loaded Ce_0.5_Zr_0.5_O_2_ was reported elsewhere to display three reduction peaks at 150 °C, 350 °C and 730 °C, attributed, respectively, to the reduction of the metal precursor, reduction of the surface/bulk Ce and further reduction of the bulk Ce [41]. Here, no peak was found at high temperatures, indicating that bulk Ce was already reduced at lower ones, together with Rh, suggesting a positive interaction between the metal and the support. This could be attributed to the homogeneous phase and morphology and the small particle size of the microemulsion-synthetized Ce_0.5_Zr_0.5_O_2_ and a homogeneous metal dispersion over the small crystals of the support. In general, the calcination temperature and the morphology obtained affected the nature of the Rh species that were deposited on the support surface. Rh-CZOm750 produced one broad peak centered at 100 °C as a result of the homogeneous reduction of Rh oxide, and Ce produced a much smaller one around 250 °C due to Ce’s being less reducible. With increasing calcination temperature, Rh-CZOm900 yielded an intense peak at 160 °C and a smaller one at 169 °C, indicating a less homogeneous reduction of the Rh-Ce system. The better reducibility of the catalyst calcined at a lower temperature could have been caused by: (i) the higher surface area and (ii) the low particle size of the CZOm750 support that provided a higher number of surface Ce sites and (iii) an increased dispersion of Rh oxide favored by the higher surface area that allowed for more interactions between the Rh species and the surface of the Ce-based oxide. On the other hand, the sample calcined at 900 °C was characterized by a lower surface area and bigger crystals that produced a less homogeneous Rh dispersion (as will be discussed in the TEM section) and thus had a lower reducibility.

Both samples displayed only one broader peak in the TPR carried out after a TPO cycle. This suggests that a more homogeneous catalyst was obtained after oxidation, where all components were reduced together. Again, the reducibility was higher in the case of the sample calcined at 750 °C, which gave a peak at a lower temperature (215 °C).

Quantification of the consumed hydrogen showed that the reducibility of the Ce support depended on the support calcination temperature. Bare supports showed similar values, suggesting that surface area was not a determining factor in hydrogen consumption but influenced mostly the reduction temperature, as CZOm750 is reduced earlier. In general, the addition of Rh increases hydrogen consumption as it causes hydrogen spillover from the metallic particle to the support.

However, the hydrogen consumption increased, decreasing the calcination temperature, which was related to the more homogeneous Rh dispersion and the small and defective CZO crystals. Not only is Rh-CZOm750 reduced at a lower temperature, it also consumes more hydrogen, reducing a higher amount of Ce. This indicates that a higher amount of oxygen is available for reduction and that its reduction is kinetically faster thanks to (i) a better Rh dispersion (as will be discussed in the TEM session), (ii) the presence of smaller CZO crystals and (iii) the higher surface area, and hence defectivity, of the CZOm750 support. In the reduction of the Ce-based support, the slow step was the reduction of bulk Ce, which was more limited for the sample calcined at 900 °C due to its larger CZO crystals [39].

**Figure 5:**
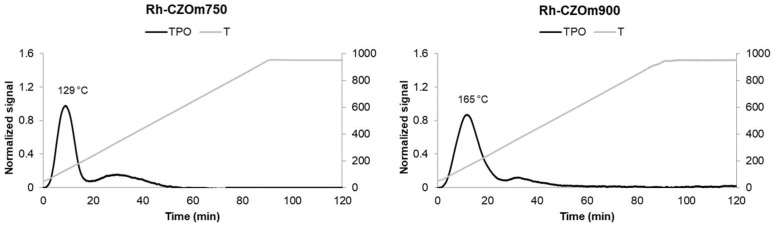
Temperature-programmed oxidation of Rh-CZOm750 and CZOm900 samples.

The TPO graphs of the impregnated samples shown in Figure 5 are very similar to those of the bare sample, as both Rh and Ce are easily oxidized at low temperatures. The catalysts showed only one main peak and a small shoulder, which suggests that both the support and the metal were mainly oxidized together, while the bulk Ce may have needed more energy to be oxidized.

#### 3.1.4. Transmission Electron Microscopy

**Figure 6:**
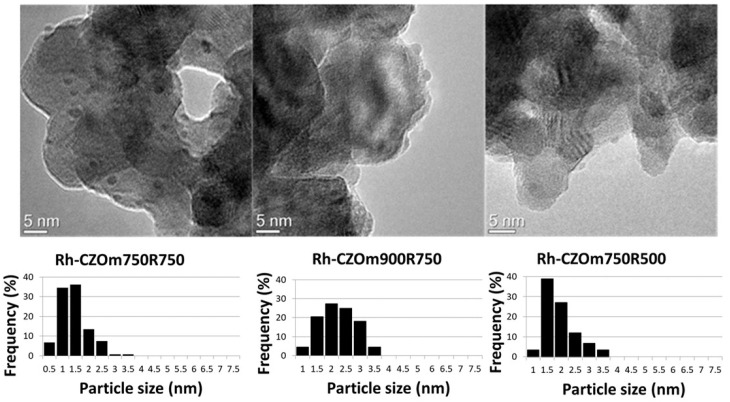
TEM images and particle size distributions of the catalysts calcined and reduced at different temperatures (Rh-CZOm750R750, Rh-CZOm900R750 and Rh-CZOm750R500).

The catalysts obtained after calcination at 750 °C or 900 °C were reduced in H_2_/N_2_ flux and analyzed by TEM. The morphologies, Rh particle dimensions, and size distributions of the catalysts treated at different calcination temperatures (Rh-CZOm750R750 and Rh-CZOm900R750) and reduction temperatures (Rh-CZOm750R750 and Rh-CZOm750R500) are shown in Figure 6.

All the samples showed the presence of small Rh particles (as confirmed by STEM, reported in Figure S6) well dispersed on CeZr nanospheres, whose shapes were templated through the microemulsion synthesis [9,33,43,44,45]. When the catalyst was calcined at 750 °C, narrow Rh size distributions were obtained, centered at 1–1.5 nm. It is worth noting that particles below 0.5 nm are not detected by the TEM used in this work, though they may be present. Higher calcination temperatures (Rh-CZOm900R750) produced a size increase in oxide particles due to sintering of the CeZr phase and slightly larger metal particles, with a broader distribution centered around 2–2.5 nm. The smaller particle size and higher surface area of the CZOm750 samples allowed for better dispersion of the rhodium nitrate precursor during the impregnation, resulting in smaller metal particles. Lowering the reduction temperature (500 °C) does not produce a particular change in the dimension of metal particles. EDS analysis conducted on the samples also confirmed the support composition to be Ce_0.5_Zr_0.5_O_2_ (Table S1).

### 3.2. Catalytic Tests

The synthesized catalysts were tested under different reaction conditions to analyze the effects of the different treatments on the catalytic performances. At first, the effect of calcination temperature was studied in oxy-reforming at 750 °C and low-temperature steam reforming (T = 350–500 °C). Then, the effect of reduction temperature was investigated.

#### 3.2.1. Effect of Calcination Temperature

The effect of the calcination temperature was investigated by comparing, in oxy-reforming and LTSR, the Rh-CZO samples calcined at 750 °C and 900 °C and reduced at 750 °C (Rh-CZOm750-R750 and Rh-CZOm900-R750, respectively).

**Figure 7:**
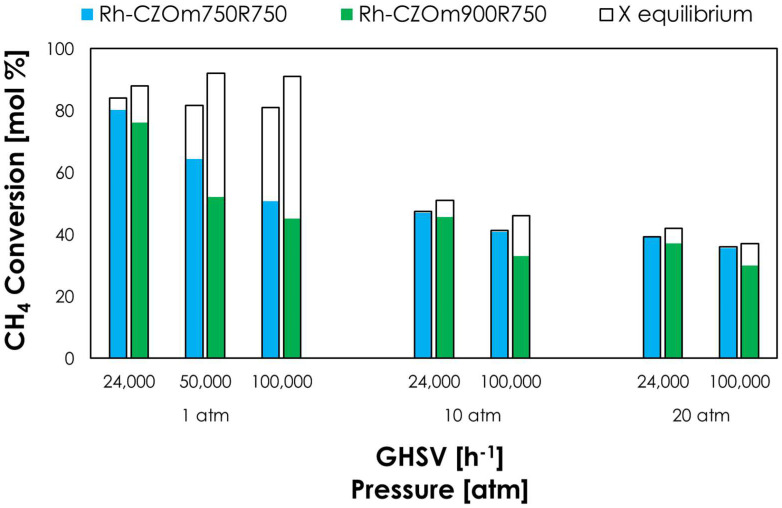
Methane conversion (colored bars) and equilibrium conversion (transparent bars) at 750 °C, S/C = 0.7, O/C = 0.2 and different pressures and GHSVs for Rh-CZOm750-R750 and Rh-CZOm900-R750.

In oxy-reforming, O_2_ is fed in a sub-stoichiometric ratio, and its total consumption by CPO occurs in the first part of the catalytic bed, followed by the conversion of the remaining methane by steam reforming. This helps to produce a smoother thermal profile than the two separate processes, as will be discussed in a following chapter. The results of the oxy-reforming tests are shown in Figure 7.

At 1 atm and 2400 h^−1^, Rh-CZOm750 provided a high methane conversion of 80%, which was close to the equilibrium value even under these kinetic-controlled conditions, and outperformed the sample calcined at 900 °C (76%). Increasing the GHSV lowered the conversion, though Rh-CZOm750 showed a higher activity even under these unfavorable conditions. Here, the equilibrium conversion of Rh-CZOm900 was higher due to the development of hot spots on the catalytic bed, as will be discussed in a dedicated paragraph. With increasing pressure, the equilibrium conversion dropped, as both reforming and CPO are favored at low pressures, though the difference between the performances of the two catalysts was lesser. Nevertheless, a lower calcination temperature allowed equilibrium to be reached at 10 and 20 atm, at both low and high GHSVs.

**Figure 8:**
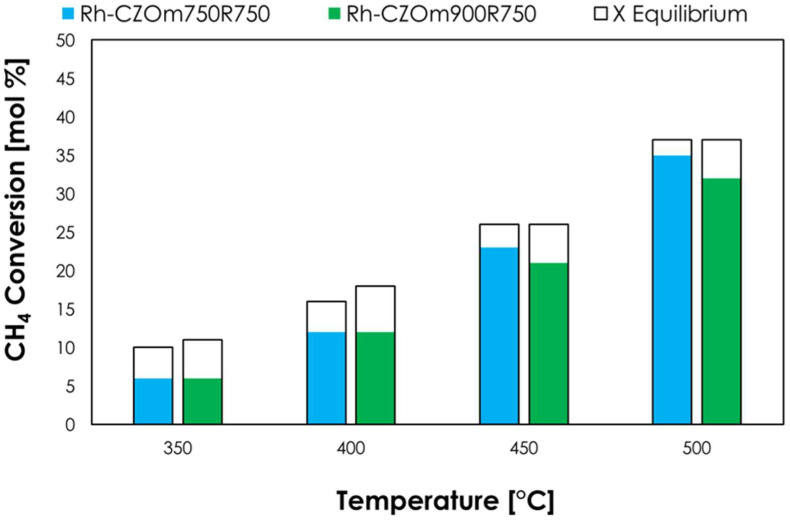
Methane conversion (colored bars) and equilibrium conversion (transparent bars) for LTSR at 1 atm, S/C = 3 and 24,000 h^−1^ for Rh-CZOm750-R750 and Rh-CZOm900-R750.

In low-temperature steam reforming (Figure 8), the two catalysts gave similar conversions at 350 and 400 °C, but Rh-CZOm750 provided higher conversions at both 450 °C (23% and 21% for Rh-CZOm750 and Rh-CZOm900, respectively) and 500 °C (35% and 32%). The better performances of Rh-CZOm750-R750 are correlated with its smaller crystal size and higher surface area (Table 2) and catalyst reducibility, which resulted in a higher Rh dispersion, as shown by TPR (where a higher amount of hydrogen was consumed and a lower reduction temperature was recorded). In particular, Ce acted as a water-dissociation site, increasing the concentration of surface OH*, which helped to increase the reaction rate in both LTSR and oxy-reforming [11,46].

#### 3.2.2. Effect of Reduction Temperature

**Figure 9:**
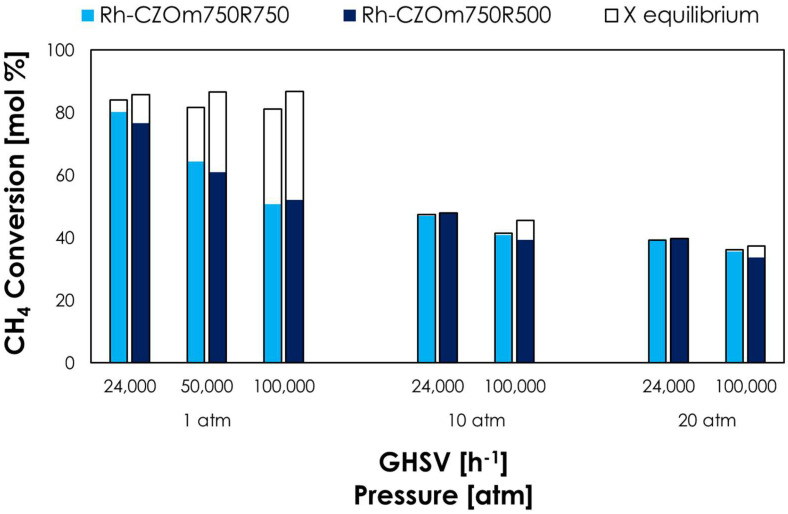
Methane conversion (colored bars) and equilibrium conversion (transparent bars) at 750 °C, S/C = 0.7, O/C = 0.2 and different pressures and GHSVs for Rh-CZOm750-R500 and Rh-CZOm750-R750.

Given the results highlighting the positive effect of lower calcination temperatures on catalytic activity, Rh-CZOm750 was reduced also at 500 °C. The results of the oxy-reforming tests carried out at 750 °C obtained at different pressures and space velocities (GHSVs) are reported in Figure 9. The highest methane conversion (76% for Rh-CZOm750-R500 and 80% for Rh-CZOm750-R750) was given at 2400 h^−1^ and 1 atm, thanks to the favorable thermodynamic and kinetic conditions. When the GHSV was raised, a drop in conversion was observed for both catalysts, far from the equilibrium value, and the catalyst performances were similar under these conditions. Increasing pressure led to a further decrease in the conversion. Under these conditions, the two catalysts reached equilibrium, showing similar performances at low GHSVs. However, when the space velocity was raised at high pressure, producing kinetic-controlled conditions, the catalyst was able to reach the equilibrium value. In general, the reduction at the higher temperature provided a slightly more active catalyst under the oxy-reforming conditions.

**Figure 10:**
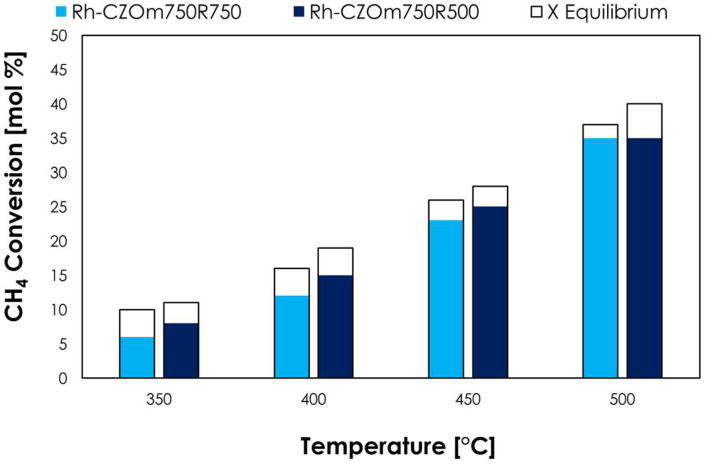
Methane conversion (colored bars) and equilibrium conversion (transparent bars) for LTSR at 1 atm, S/C = 3 and 24,000 h^−1^ for Rh-CZOm750-R500 and Rh-CZOm750-R750.

The catalysts were also tested in low-temperature steam reforming at 1 atm, 24,000 h^−1^, S/C = 3 and different temperatures (Figure 10). In this case, the performances of the catalysts were similar at 750 °C, while a slightly higher methane conversion was observed below 400 °C for the catalyst reduced at a lower temperature. In general, the two catalytic systems showed similar performances in methane conversion to syngas.

The similar behavior of the two catalysts is related to the similar Rh particle size and distribution observed by TEM. The slight differences observed are ascribable to small variations that arise when catalysts are submitted to oxy-reforming reaction conditions. In fact, the sample reduced at a lower temperature was submitted to a hydrogen-rich atmosphere when the reaction produced H_2_ at 750 °C, which may have led to a slight transformation of the catalyst. On the other hand, the sample reduced at a higher temperature was more stable under these conditions, as the reduction had been already conducted at 750 °C.

#### 3.2.3. Oxy-Reforming Temperature Profile

**Figure 11:**
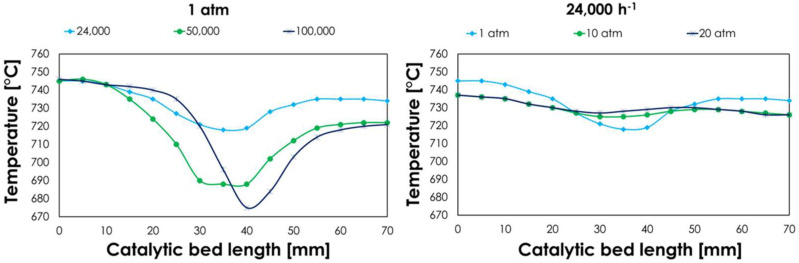
Temperature profile of the catalytic bed for Rh-CZOm750R750 at different pressures (left) and GHSVs (right) obtained with a sliding thermocouple.

In hydrogen production by classical steam reforming or catalytic partial oxidation, the development of important endothermic or exothermic profiles inside the reactor affect catalytic performance in terms of activity, stability, and equilibrium conversion [47,48,49]. For instance, a very active steam reforming catalyst would consume a high amount of methane and thus a high amount of heat, causing a sharp decrease in temperature over the catalytic bed. Hence, it would reduce the equilibrium conversion and catalyst activity, as steam reforming is favored at high temperatures. This makes catalyst comparison difficult when different conversions are involved due to more or less active samples. The same concept applies to exothermic profiles developed during CPO. This process follows a direct or indirect route [50]. In the case of Rh/CeO_2_-ZrO_2_, an indirect route has been reported that involves fast and exothermic oxidation of methane in the first part of the catalytic bed, which produces CO_2_ and H_2_O [51]. These then consume methane through dry or steam reforming, giving H_2_ and CO in the second part of the catalytic bed. The oxy-reforming process studied here allows for the mitigation of the temperature increases or decreases that characterize the distinct processes by coupling them, exploiting the heat developed in the oxidation reactions to feed the endothermic steam reforming. This can be pursued with fine-tuning of reaction conditions and catalyst properties. For instance, Maestri et al. observed a negligible change in the temperature profile when they carried out CPO with a high amount of oxygen (O/C = 1) and the addition of steam [52]. However, they added more oxygen and a slightly lower amount of steam compared to this work (S/C = 0.5) and performed the tests on a Rh/Al_2_O_3_ catalyst. Under these conditions, oxidation was favored over the first part of the bed, where methane dissociation was the limiting step, and the oxygen mass transfer governed the formation of the main oxidizer *OH. Here, oxygen consumption was very fast and resulted in hot spots in the first part of the catalytic bed, then was followed by a decrease in reaction temperature in the second part of the catalytic bed caused by steam reforming [53]. The results obtained in the present work show that feeding an optimized amount of steam and oxygen (S/C = 0.7 and O_2_/C = 0.2) allows the ΔT of the process to be reduced. Here, a temperature drop of only 20 °C was observed over the catalytic bed for Rh/CZOm750R750 at 2400 h^−1^ and 1 atm (Figure 11).

Moreover, no exothermic peak was shown, indicating that the consumption of oxygen by oxidative exothermic processes was compensated by the occurrence of the endothermic steam reforming. This not only permitted an increase in the energy efficiency of the process but also made catalyst comparison more reliable and less dependent on the developed temperature profile. Nevertheless, the temperature profile was dependent on the amount of converted methane, which was affected by the operative conditions and the thermal exchange properties of the catalyst. Increasing GHSV lowered the percentage conversion but increased the methane flux, and hence the total consumed methane, resulting in a sharper drop in temperature over the catalytic bed and a downward shift of the peak, which is in line with the reported effect of flow rate on the temperature of the gas phase [53,54]. On the other hand, experimental and equilibrium methane conversions decreased at high pressures, smoothening the profile.

**Figure 12:**
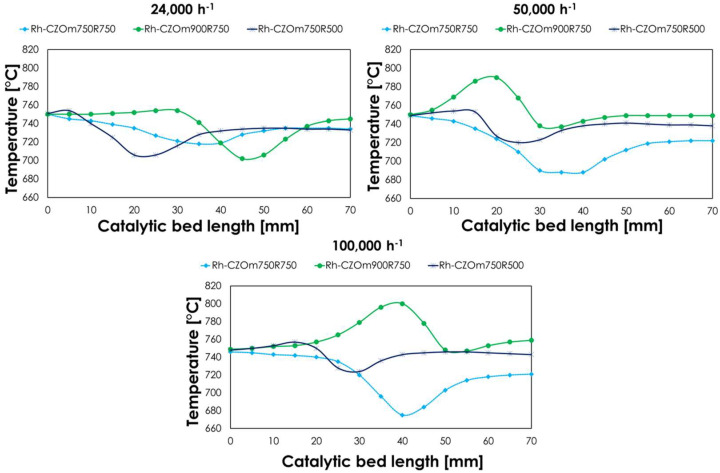
Temperature profile of the catalytic bed of the investigated catalysts at T_oven_ = 750 °C, 1 atm, S/C = 0.7, O2/C = 0.2 and 24,000 h^−1^ (left), 50,000 h^−1^ (center) or 100,000 h^−1^ (right) obtained with a sliding thermocouple.

The temperature profile is also dependent on the catalyst’s properties. Comparing the investigated catalysts, the biggest differences arose at 1 atm and different GHSVs, as shown in Figure 12.

Interestingly, Rh-CZOm750R750, the most active catalyst under these conditions, provided the lower ΔT at a low space velocity. At higher GHSVs, it showed the lowest recorded temperature, while exothermic peaks arose in the first part of the catalytic bed for the other catalysts, followed by endothermic ones that were due to the distinct occurrences of the fast oxidations in the first part of the catalytic bed, followed by steam reforming, as characteristics for the processes dominated by methane oxidation reactions [11,52,53,55]. On the other hand, the profile recorded for Rh/CZOm750R750 suggests that oxidation and reforming reactions occur concurrently, enhancing local thermal exchange, and giving low ΔT, regardless of the higher activity, with a lower temperature at the outlet. This is indicative of a higher promotion of steam reforming, confirmed also by the high conversion provided in the low-temperature steam reforming tests with this catalyst, which anticipated methane consumption by this process to a part of the catalytic bed where oxidation predominantly occurs. The heat released by oxidation is unaffected by the surface oxidation rate because oxygen consumption is governed by oxygen external mass transfer [53]. On the other hand, heat consumption is greatly affected by the local promotion of steam reforming. In general, an interesting feature of oxy-reforming compared to sole SR or CPO is that higher conversions are matched with better heat exchange and less pronounced thermal profiles, which allows better heat exchange and more reliable comparison of active catalysts.

#### 3.2.4. Catalyst Stability

**Figure 13:**
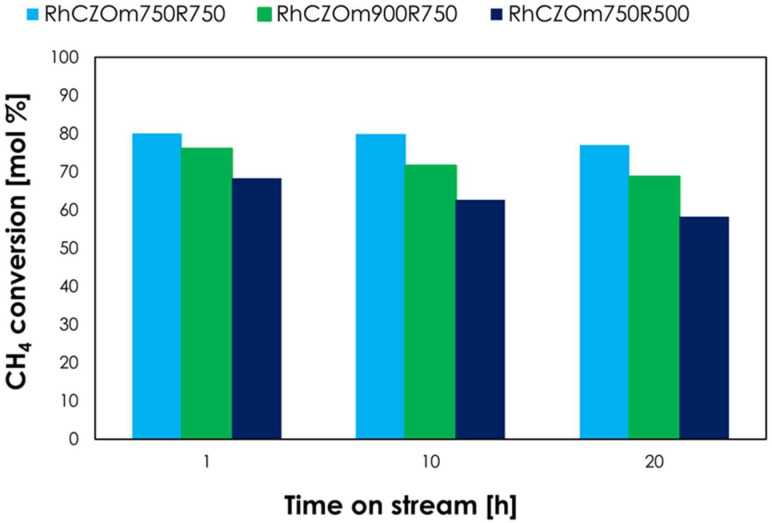
Methane conversion over time on stream in repeated tests carried out at 750 °C, 24,000 h^−1^ and 1 bar for the investigated catalysts.

In order to assess the catalyst stability, “return” tests were carried out periodically after the screening of different conditions (Figure 13). These consisted of the repetition of the first test and allowed us to evaluate catalyst deactivation by comparing the obtained methane conversion with that obtained with the as-prepared catalyst. For catalysts reduced at 750 °C, only a slight conversion drop was observed after many hours of operation. In particular, the best performances were given by Rh-CZOm750-R750, which showed a conversion decrease of only 3% after 20 h of operation. The sample calcined at 750 °C and reduced at 750 °C lost 4% of conversion after just 10 h and 7% after 20 h of operation. Decreasing the reduction temperature provided a lower stability, as Rh-CZOm750-R500 lost 10% of conversion after 22 h, though this catalyst was outperformed by its analogues reduced at 750 °C, regardless of the interesting methane conversions displayed in the tests under different reaction conditions. The good performances of Rh-CZOm750-R750 are linked to the homogenous Rh dispersion and high surface area provided by the high reduction and low calcination temperatures, respectively. In particular, these resulted in better interactions of Rh with the small Ce nanoparticles in this sample, as highlighted by TPR, where a higher percentage of Ce was reduced and the reduction temperature was lower. Carbon formation on the catalyst surface was avoided thanks to the oxygen-storage capacity and mobility of the support, which is able to oxidize the carbon that is eventually formed, slowing down deactivation. To shed further light on the catalysts’ stabilities, the samples were analyzed after the reaction (denoted as “’spent’ samples”).

#### 3.2.5. Characterization of the Spent Catalysts

The catalysts were characterized after the reactivity tests investigating their morphological properties and the presence of carbon deposited on the surface by XRD, nitrogen physisorption and Raman analyses.

**Figure 14:**
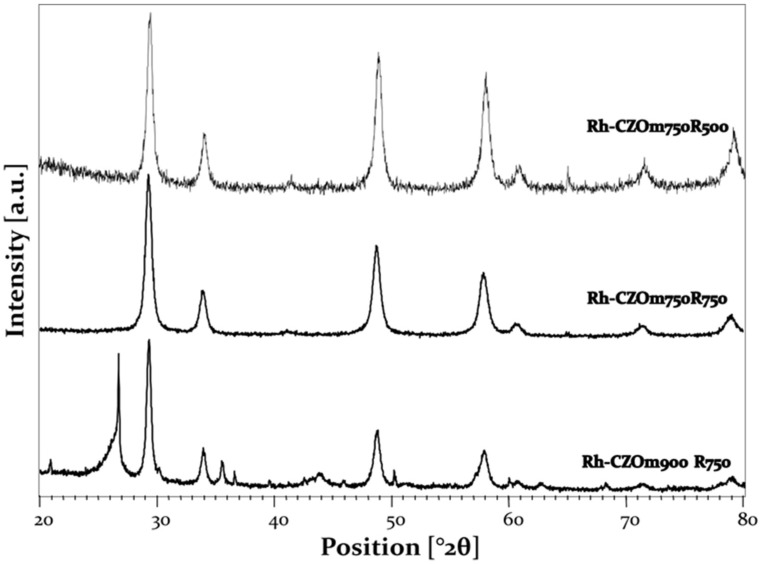
XRD analysis of the spent catalysts (from top to bottom) Rh-CZO-m750R500, CZO-m750R750 and Rh-CZO-m900R750.

The diffraction pattern of the Ce_0.5_Z_r0.5_O_2_ mixed oxide phase was observed in the XRD of the used catalysts, indicating that no phase segregation occurred during the reactivity test (Figure 14). In the case of the sample calcined at 900 °C, the presence of carbon was observed by the reflection at 26° 2θ, which was formed as a sub-product during the reactivity tests [56]. When the average crystallite size of the samples was calculated with the Scherrer equation, all the samples showed similar dimensions of crystallites for Rh-CZOm750-R750 and Rh-CZOm900-R750 (11 and 20 nm) compared to the fresh samples (Rh-CZOm750 8 nm; CZOm900 20 nm). On the other hand, the crystallite average size doubled to 16 nm for Rh-CZOm750-R500. This shows that sintering of the support particles had occurred during the harsh reaction conditions in the latter case, which is consistent with the lower activity and stability of this catalyst.

**Figure 15:**
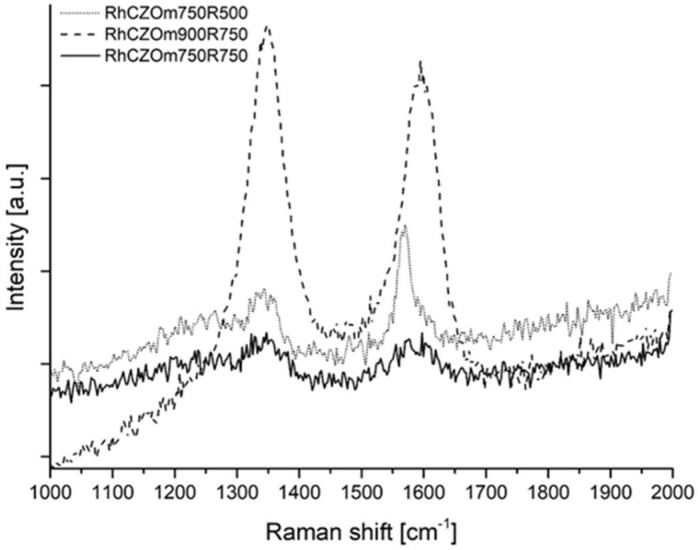
Raman analysis of the spent catalysts Rh-CZO-m750R500, CZO-m750R750 and Rh-CZO-m900R750.

Catalyst stability can also be hindered by the formation of carbon over the catalytic surface, a side reaction that is favored under oxy-reforming conditions [9]. When carbon is formed, it covers the surface of the catalyst, leading to removal of the active phase from the reaction environment, resulting in deactivation. The presence of carbon over the catalyst, already detected by XRD for the sample calcined at a higher temperature, was further investigated by Raman analysis, in which two bands related to carbon were easily detected at 1350 and 1580 cm^−1^. These bands are called D-bands and G-bands. G-bands are related to the stretching of sp^2^ carbon bonds in ordered graphite, while D-bands are due to the vibrations of disordered carbon atoms. Figure 15 shows the Raman analysis carried out on the used samples.

Carbon presence was evident on the Rh-CZOm750-R500 sample, and its occurrence can be addressed, together with the sintering of the support, for the lower stability of this catalyst. Carbon was also formed on the catalyst calcined at 900 °C but was barely observable on Rh-CZOm750-R500 (being detected in only one of the multiple Raman analyses conducted on this sample, the one shown in Figure 15).

This is consistent with the results of the stability tests that demonstrated a high stability for this sample. This phenomenon can be attributed to the high metal dispersion and interaction between Ce and Rh, also demonstrated by the higher hydrogen consumption in TPR for this catalyst. The higher interaction and the high metal dispersion on a larger surface area favored the exchange of mobile oxygen from the reducible Ce-based support to the Rh active phase where carbon was produced, oxidizing it efficiently and preserving catalytic activity.

## 4. Conclusions

Different Rh-based catalysts were synthesized by impregnation of the metal over a Ce_0.5_Zr_0.5_O_2_ support obtained by inverse microemulsion synthesis. The supports were calcined at 750 °C or 900 °C and the catalysts were reduced at 750 °C or 500 °C and fully characterized by means of XRD, nitrogen physisorption, TPR and TEM. Calcination at 900 °C provided a lower surface area and consequently lower reducibility. On the other hand, calcining the support at 750 °C obtained well-dispersed Rh particles with higher reducibility and hydrogen consumption during TPR. The catalysts were tested in methane oxy-reforming at 750 °C, feeding sub-stoichiometric oxygen and steam together with methane. In an integrated vision, the oxygen needed for the oxy-reforming process comes from an electrolyzer, where it is produced together with green hydrogen. The catalyst calcined at 750 °C and reduced at the same temperature provided not only high methane conversion, but also the best stability thanks to a good Rh-CeZr oxide interaction that disfavored carbon formation. It also displayed the smoothest thermal profile, thanks to the anticipation of the steam reforming given by high reducibility of the Rh-CeZr oxide system which increased methane dissociation and the concentration of surface OH* and allowed the process to run with less sharp temperature gradients over the catalytic bed.

## Figures and Tables

**Table 1:** Operative conditions employed in this work.

Operative Parameter	Low-Temperature Steam Reforming	Oxy-Reforming
Temperature (°C)	500; 450; 400; 350	750
Pressure (atm)	1	1; 10; 20
GHSV (h^−1^)	24,000	24,000; 50,000; 100,000
Gas feed composition (vol. %) (CH_4_, O_2_, H_2_O)	25; 75; 0	52.04; 11.20; 36.75
Steam-to-carbon ratio (S/C)	3	0.71
Oxygen-to-carbon ratio (O_2_/C)	0	0.21

**Table 2:** Surface area, pore volume and average pore diameter of the CZO samples calcined at 900 (CZOm900) and 750 °C (CZOm750).

Catalyst	Surface Area (m^2^/g)	Pores Volume (cm^3^/g)	Avg. Pore Diameter (nm)
CZOm750	54.1	0.09	4.8
CZOm900	14.5	0.08	19.3
Rh-CZOm750	34.6	0.07	5.6
Rh-CZOm900	11.5	0.06	19.7

**Table 3:** Hydrogen consumption during temperature-programmed reduction for bare supports and impregnated samples.

Catalyst	H_2_ cons. in TPR1 (mmol/g)	% Ce Reduced	H_2_ cons. in TPR2 (mmol/g)	% Ce Reduced
CZOm750	1.49	55	1.48	56
CZOm900	1.41	60	1.44	59
Rh-CZOm750	1.91	82	2.03	89
Rh-CZOm900	1.76	74	1.67	69

## Data Availability

The data used in this study are contained within the article or the Supplementary Materials.

## Supplementary Materials

The following supporting information can be downloaded at: www.mdpi.com/article/10.3390/nano13010053/s1, Figure S1: XRD of the samples calcined at different temperatures (500–1100 °C); Figure S2: Isothermal linear plots of bare supports; Figure S3: Pore size distribution of bare supports; Figure S4: Isothermal linear plot of Rh-CZO catalysts; Figure S5: Pore size distribution of Rh-CZO catalysts; Figure S6: STEM electron image of Rh nanoparticles; Table S1: Ce and Zr atomic percentages in CZO and Rh-CZO catalysts measured by EDS microanalysis.
