# Supplementary material for: H2 Production by Methane Oxy-Reforming: Effect of Catalyst Pretreatment on the Properties and Activity of Rh-Ce0.5Zr0.5O2 Synthetized by Microemulsion
Source: Nanomaterials (Basel). 2022 Dec 22;13(1):53. doi: 10.3390/nano13010053 (PMC9823839; doi:10.3390/nano13010053)
Supplement: Supplementary file 1 [file nanomaterials-13-00053-s001.zip › nanomaterials-2089532-supplementary.pdf]

nanomaterials

MDPI

Supporting Information

H_2 Production by Methane Oxy-Reforming: Effect of Catalyst Pretreatment on the Properties and Activity of Rh-$Ce_{0.5}Zr_{0.5}O_2$ Synthetized by Microemulsion

Jacopo De Maron [1,2], Rodolfo Mafessanti [1], Pio Gramazio [1,2], Elisabetta Orfei [1,2], Andrea Fasolini [1,2,*] and Francesco Basile [1,2,*]

1 "Toso Montanari" Industrial Chemistry Department, Alma Mater Studiorum, Università di Bologna, 40136 Bologna, Italy
2 Center for Chemical Catalysis—C3, Alma Mater Studiorum, Università di Bologna, 40136 Bologna, Italy
* Correspondence: andrea.fasolini@unibo.it (A.F.); f.basile@unibo.it (F.B.)

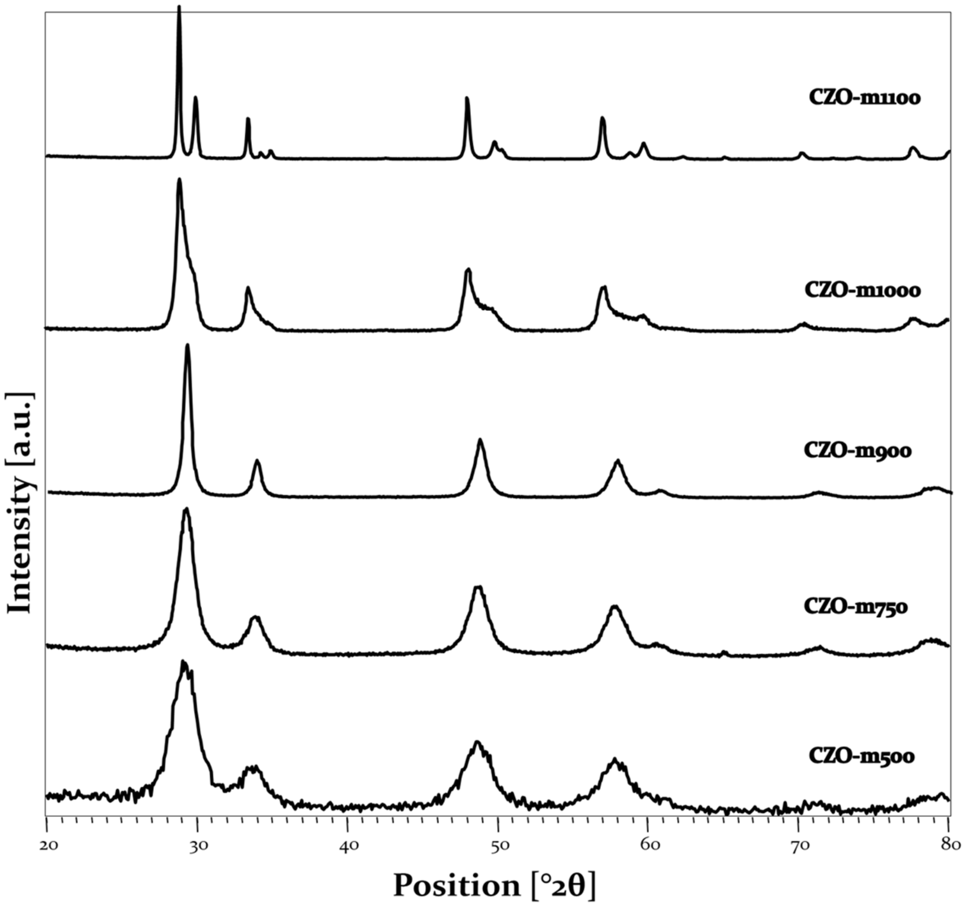

2

Figure S1. XRD of the CZO samples calcined at different temperatures: 500°C (CZO-m500), 750°C (CZO-m750), 900°C (CZO-m900), 1000°C (CZO-m1000) and 1100°C (CZO-m1100).

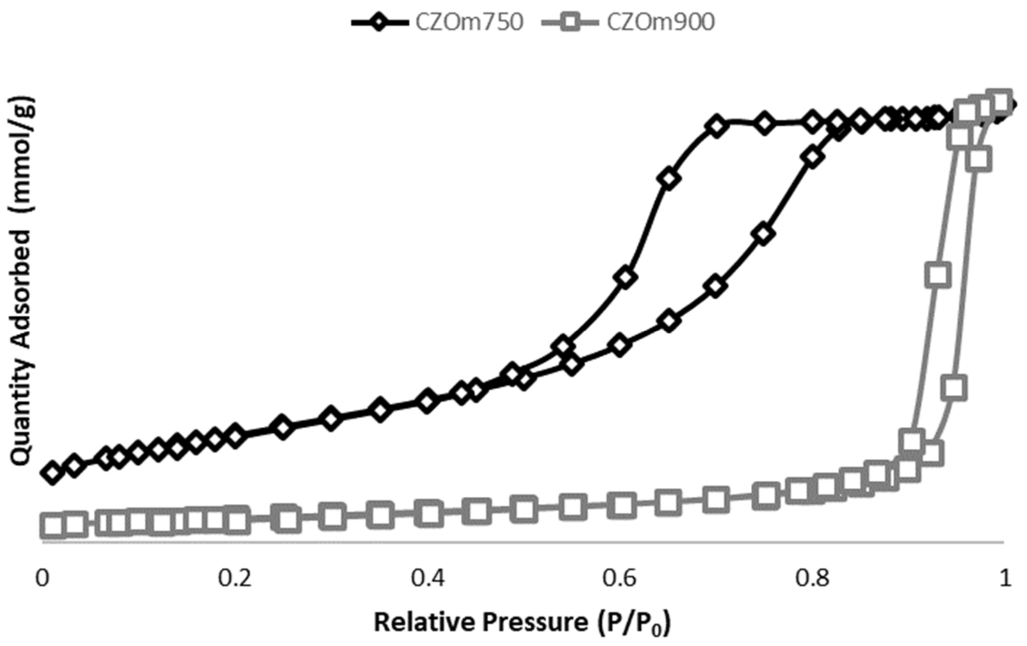

Figure S2. Isothermal linear plots of the CZOm750 (black) and CZOm900 (grey) samples.

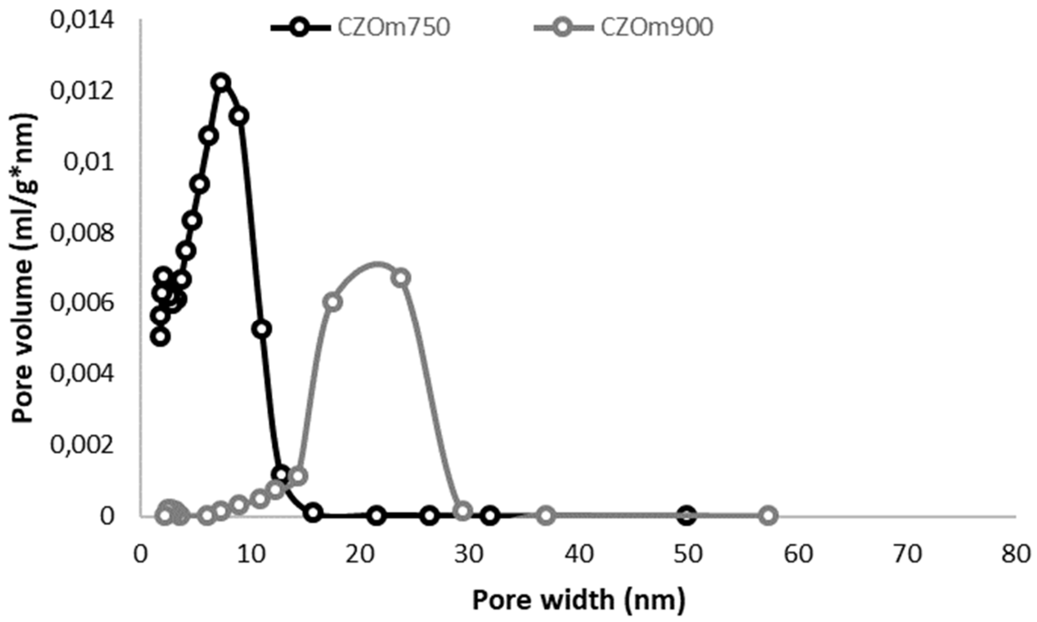

Figure S3. Pore size distribution of the CZOm750 (black) and CZOm900 (grey) samples.

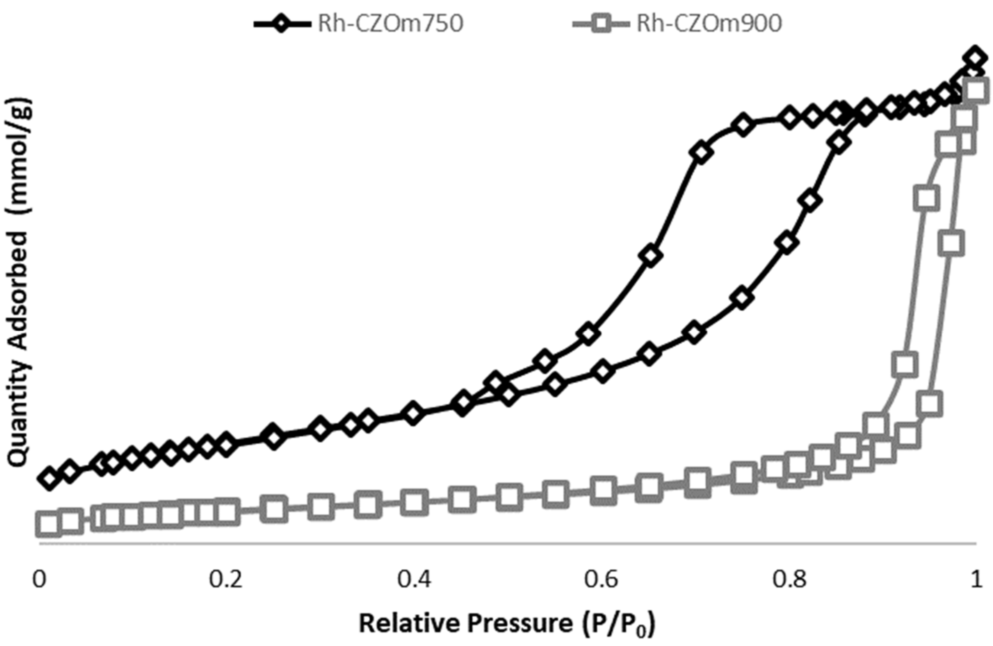

Figure S4. Isothermal linear plots of the Rh-CZOm750 (black) and Rh-CZOm900 (grey) samples.

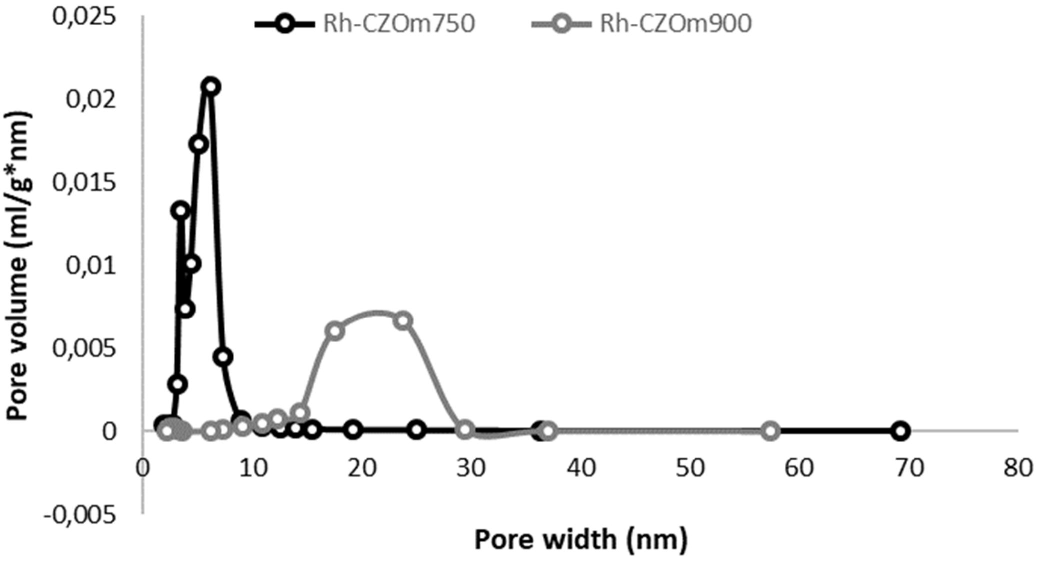

Figure S5. Pore size distribution of the Rh-CZOm750 (black) and Rh-CZOm900 (grey) samples.

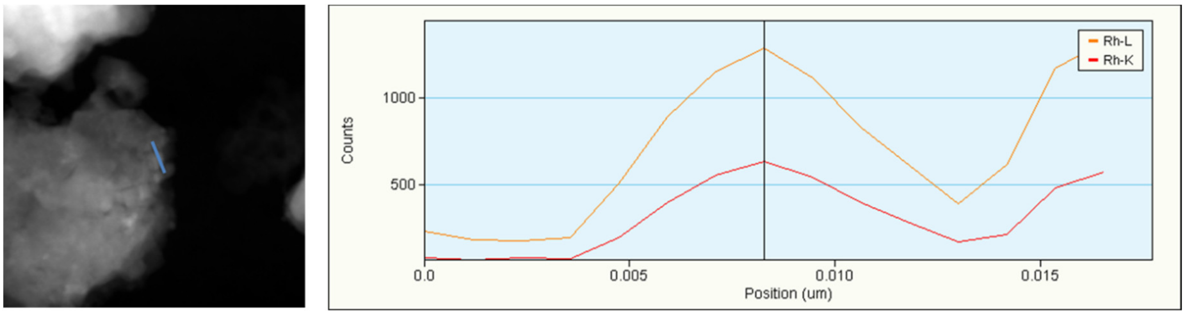

Figure S6. STEM analysis of the Rh-CZOm900-R750 sample confirming that the displayed nanoparticles are metallic Rh crystals.

Table S1. Atomic percentage between Ce and Zr in the Rh-CZO samples as determined by TEM-EDS. Only Ce and Zr were considered in the analysis.

	**% Ce**	**% Zr**
Area 1	51.1	48.9
Area 2	52.0	48.0
Area 3	50.1	49.9
Area 4	50.4	49.6
